# Risk factors and epidemiology of human T-lymphotropic virus types 1 and 2 in US blood donors

**DOI:** 10.1186/1742-4690-12-S1-P84

**Published:** 2015-08-28

**Authors:** Farnaz Vahidnia, Susan L Stramer, Debra A Kessler, David E Krysztof, Roger Y Dodd, Ed Notari, Simone Glynn, Brian Custer

**Affiliations:** 1 Blood Systems Research Institute, San Francisco, CA, USA; 2 American Red Cross, Rockville, MD, USA; 3 New York Blood Center, New York, NY, USA; 4 National Heart, Lung and Blood Institute, Bethesda, MD, USA

## 

Blood donations in the US are routinely screened for markers of human T-lymphotropic virus (HTLV). Seroprevalence and risk factors associated with HTLV-1 and -2 infection from the US Retrovirus Epidemiology Donor Study (REDS-II) Transfusion-Transmitted Viral Infection (TTVI) Rate and Risk Factor Study are reported. Among 14,809,334 blood donations screened during 2011-2012, 516 HTLV confirmed seropositive cases were identified, with an overall prevalence of 3.5 infections per 1,0 donations (95% CI: 3.2-3.8). A case-control study of risk factors from 90 donors with serology-confirmed HTLV-1 infection, 102 with HTLV-2 (cases), and 1,587 donors with false-positive results (controls) was conducted. Frequencies and adjusted odds ratios (AORs) from separate multivariable logistic regression analyses for HTLV-1 and -2 cases compared to controls are reported. Mean age was 48.0 (SD: 12.4), 52.3 (SD: 11.0) and 41.7 (SD: 15.7) years for HTLV-1, HTLV-2 cases and controls, respectively. Being a first-time donor, older, non-white, non-Hispanic female were significant demographic factors associated with both infections. HTLV-1 cases were more likely than controls to be Black (AOR: 13.3, 95% CI: 6.1-29.2), born outside of the US (AOR: 8.6, 95% CI: 4.0-18.4), have migrated (family or self) from an endemic area (AOR: 1.8, 95% CI: 1.2-2.7), report sex with an IDU (AOR: 10.9, 95% CI: 3.6-33.4) or have multiple partners (AOR: 3.1, 95% CI: 1.5-6.1). HTLV-2 cases were more likely to be Black (AOR: 15.4, 95% CI: 6.9-34.3), Native Americans (AOR: 10.7, 95% CI: 1.5-77.0), report sex with an IDU (AOR: 27.2, 95% CI: 9.7-75.8) or have multiple partners (AOR: 2.9, 95% CI: 1.5-5.7). US blood donors with HTLV-1 or -2 infection present with the known risk factors. Migration from endemic areas is mainly associated with HTLV-1 infection, while HTLV-2 is associated with Native American donors. Sexual risk behavior and IDU continue to be risk factors for both viruses.

